# Supplementary material for: Bayesian refinement of protein structures and ensembles against SAXS data using molecular dynamics
Source: PLoS Comput Biol. 2017 Oct 18;13(10):e1005800. doi: 10.1371/journal.pcbi.1005800 (PMC5662244; doi:10.1371/journal.pcbi.1005800)
Supplement: S1 Archive — (BZ2) [file pcbi.1005800.s011.tar.bz2 › gromacs_4.62_bayesian_saxs_refi/share/html/online/g_tcaf.html]

g\_tcaf


| g\_tcaf Main Table of Contents |

**VERSION 4.6  
Sat 19 Jan 2013**

---

### Description

g\_tcaf computes tranverse current autocorrelations.
These are used to estimate the shear viscosity, η.
For details see: Palmer, Phys. Rev. E 49 (1994) pp 359-366.

Transverse currents are calculated using the
k-vectors (1,0,0) and (2,0,0) each also in the y- and z-direction,
(1,1,0) and (1,-1,0) each also in the 2 other planes (these vectors
are not independent) and (1,1,1) and the 3 other box diagonals (also
not independent). For each k-vector the sine and cosine are used, in
combination with the velocity in 2 perpendicular directions. This gives
a total of 16\*2\*2=64 transverse currents. One autocorrelation is
calculated fitted for each k-vector, which gives 16 TCAFs. Each of
these TCAFs is fitted to f(t) = exp(-v)(cosh(Wv) + 1/W sinh(Wv)),
v = -t/(2 τ), W = sqrt(1 - 4 τ η/ρ k^2), which gives 16 values of τ
and η. The fit weights decay exponentially with time constant w (given with -wt) as exp(-t/w), and the TCAF and
fit are calculated up to time 5\*w.
The η values should be fitted to 1 - a η(k) k^2, from which
one can estimate the shear viscosity at k=0.

When the box is cubic, one can use the option -oc, which
averages the TCAFs over all k-vectors with the same length.
This results in more accurate TCAFs.
Both the cubic TCAFs and fits are written to -oc
The cubic η estimates are also written to -ov.

With option -mol, the transverse current is determined of
molecules instead of atoms. In this case, the index group should
consist of molecule numbers instead of atom numbers.

The k-dependent viscosities in the -ov file should be
fitted to η(k) = η\_0 (1 - a k^2) to obtain the viscosity at
infinite wavelength.

**Note:** make sure you write coordinates and velocities often enough.
The initial, non-exponential, part of the autocorrelation function
is very important for obtaining a good fit.

### Files

| option | filename | type | description |
| --- | --- | --- | --- |
| **-f** | traj.trr | Input | Full precision trajectory: trr trj cpt |
| **-s** | topol.tpr | Input, Opt. | Structure+mass(db): tpr tpb tpa gro g96 pdb |
| **-n** | index.ndx | Input, Opt. | Index file |
| **-ot** | transcur.xvg | Output, Opt. | xvgr/xmgr file |
| **-oa** | tcaf\_all.xvg | Output | xvgr/xmgr file |
| **-o** | tcaf.xvg | Output | xvgr/xmgr file |
| **-of** | tcaf\_fit.xvg | Output | xvgr/xmgr file |
| **-oc** | tcaf\_cub.xvg | Output, Opt. | xvgr/xmgr file |
| **-ov** | visc\_k.xvg | Output | xvgr/xmgr file |

### Other options

| option | type | default | description |
| --- | --- | --- | --- |
| **-[no]h** | bool | no | Print help info and quit || **-[no]version** | bool | no | Print version info and quit || **-nice** | int | 19 | Set the nicelevel || **-b** | time | 0 | First frame (ps) to read from trajectory || **-e** | time | 0 | Last frame (ps) to read from trajectory || **-dt** | time | 0 | Only use frame when t MOD dt = first time (ps) || **-[no]w** | bool | no | View output .xvg, .xpm, .eps and .pdb files || **-xvg** | enum | xmgrace | xvg plot formatting: xmgrace, xmgr or none || **-[no]mol** | bool | no | Calculate TCAF of molecules || **-[no]k34** | bool | no | Also use k=(3,0,0) and k=(4,0,0) || **-wt** | real | 5 | Exponential decay time for the TCAF fit weights || **-acflen** | int | -1 | Length of the ACF, default is half the number of frames || **-[no]normalize** | bool | yes | Normalize ACF || **-P** | enum | 0 | Order of Legendre polynomial for ACF (0 indicates none): 0, 1, 2 or 3 || **-fitfn** | enum | none | Fit function: none, exp, aexp, exp\_exp, vac, exp5, exp7, exp9 or erffit || **-ncskip** | int | 0 | Skip this many points in the output file of correlation functions || **-beginfit** | real | 0 | Time where to begin the exponential fit of the correlation function || **-endfit** | real | -1 | Time where to end the exponential fit of the correlation function, -1 is until the end |

---

http://www.gromacs.org  
gromacs@gromacs.org
